# Gene Expression Regulation and Secretory Activity of Mesenchymal Stem Cells upon In Vitro Contact with Microarc Calcium Phosphate Coating

**DOI:** 10.3390/ijms21207682

**Published:** 2020-10-16

**Authors:** Larisa Litvinova, Kristina Yurova, Valeria Shupletsova, Olga Khaziakhmatova, Vladimir Malashchenko, Egor Shunkin, Elena Melashchenko, Natalia Todosenko, Marina Khlusova, Yurii Sharkeev, Ekaterina Komarova, Maria Sedelnikova, Igor Khlusov

**Affiliations:** 1 Center for Immunology and Cellular Biotechnology, Immanuel Kant Baltic Federal University, 236000 Kaliningrad, Russia; kristina_kofanova@mail.ru (K.Y.); VShupletsova@mail.ru (V.S.); hazik36@mail.ru (O.K.); VlMalashchenko@kantiana.ru (V.M.); egor.shunkin@gmail.com (E.S.); lena.melashchenko17@mail.ru (E.M.); Tod_89@mail.ru (N.T.); khlusov63@mail.ru (I.K.); 2 Department of Pathophysiology, Siberian State Medical University, 634050 Tomsk, Russia; uchsovet@ssmu.ru; 3 Laboratory of Physics of Nanostructured Biocomposites, Institute of Strength Physics and Materials Science, SB RAS, 634055 Tomsk, Russia; sharkeev@ispms.ru (Y.S.); katerina@ispms.ru (E.K.); smasha5@yandex.ru (M.S.); 4 Research School of High-Energy Physics, Tomsk Polytechnic University, 634055 Tomsk, Russia; 5 Research School of Chemistry and Applied Biomedical Sciences, Tomsk Polytechnic University, 634050 Tomsk, Russia; 6 Department of Morphology and General Pathology, Siberian State Medical University, 634050 Tomsk, Russia

**Keywords:** human adipose tissue, stem cell culture, cytokine/chemokine genes, osteogenic genes, microarc oxidation technique, technological coating properties, correlations

## Abstract

The manufacture of biomaterial surfaces with desired physical and chemical properties that can directly induce osteogenic differentiation without the need for biochemical additives is an excellent strategy for controlling the behavior of mesenchymal stem cells (MSCs) in vivo. We studied the cellular and molecular reactions of MSCs to samples with a double-sided calcium phosphate (CaP) coating and an average roughness index (Ra) of 2.4–4.6 µm. The study aimed to evaluate the effect of a three-dimensional matrix on the relative mRNA expression levels of genes associated with the differentiation and maturation of MSCs toward osteogenesis (*RUNX2*, *BMP2*, *BMP6*, *BGLAP*, and *ALPL*) under conditions of distant interaction in vitro. Correlations were revealed between the mRNA expression of some osteogenic and cytokine/chemokine genes and the secretion of cytokines and chemokines that may potentiate the differentiation of cells into osteoblasts, which indicates the formation of humoral components of the extracellular matrix and the creation of conditions supporting the establishment of hematopoietic niches.

## 1. Introduction

Osteoimmunology is a modern, rapidly developing field [1] of regenerative medicine. However, little is known about the interactions of bone and immune cells and the roles of secreted mediators [2], and the available data are contradictory [2,3].

On the one hand, inflammation is a protective reaction to tissue damage, and the optimal strength and duration of inflammation is necessary to prepare the tissue for subsequent recovery [2]. However, inflammatory cells and their mediators are not always required for tissue regeneration; their absence can even accelerate wound healing [3]. This contradiction may be explained by the observation that mesenchymal stem cells (MSCs) and their derivatives (osteoblasts, chondroblasts, and fibroblasts) can gradually produce both inflammatory and osteogenic cytokines within 7–14 days after the injury and during the subsequent healing stages [4]. The secretory activity of MSCs is also manifested when induced by exposure to substrates with calcium phosphate (CaP) coatings [5].

On the other hand, numerous genes/signaling pathways controlling the osteogenic differentiation of MSCs, secretion of regulatory proteins and mineralization (calcification) of the extracellular matrix (ECM) are known and persistently studied. These genes/signaling pathways include those induced by contact with sources of calcium and phosphorus, such as Runt-related transcription factor 2 (Runx2), alkaline phosphatase (ALP), osteocalcin (OCN), osteopontin (OPN), bone morphogenetic proteins (BMPs), mammalian homolog of Drosophila wingless (Wnt) ligands/β-catenin, the Ca^2+^-sensing receptor (CaR), and Ca^2+^/protein kinase C [6,7,8]. However, knockdown of individual genes/transcription factors only partially limits MSC proliferation and differentiation [9,10]. Occasionally, recombinant human bone morphogenetic proteins (BMPs) alone cannot induce MSCs to undergo ECM mineralization in vitro [11]. In addition, elevated activity of the osteoblast marker ALP or an increased concentration of OCN is not always necessary for ECM mineralization in human cells [12].

Apparently, the molecular foundations underlying the osteogenic differentiation and maturation of MSCs are incompletely defined. For example, information has recently been accumulating on the osteomodulatory effects of inflammatory signaling molecules [13,14]. The surface properties of implants affect the expression of genes and molecules mediating the intercellular signaling and differentiation of osteoblasts [15] to promote bone substitute material-mediated regeneration [16]. These processes are largely dependent on the solubility [8] and surface topography [17] of the CaP biomaterial.

Microarc oxidation (MAO) or plasma electrolytic oxidation (PEO) is one the appropriate method to deposit CaP coatings for biomedical applications [18,19,20]. We found previously ECM mineralization of 21-day MSC culture both on rough surface [21] and on plastic around the microarc CaP-coated titanium (Ti) samples [22]. ALP and OCN expression [21] and secretion [23] accelerated by in vitro contact with microarc CaP coating. At the same time, microarc CaP coating effect on osteogenic gene expression in MSCs is still unclear from available publications, e.g., [20,24,25]. 

In this context, this work aimed to study the possibility of regulating osteogenic gene expression mediated by the intercellular cytokine/chemokine signaling of MSCs induced by in vitro contact with a scaffold-like CaP coating with a rough surface deposited on Ti substrates by MAO method.

## 2. Results and Discussion

### 2.1. Cell Viability and Cellular Immunophenotype

In a standard culture medium without osteogenic differentiation additives, the viability of adherent fibroblast-like cells of human adipose tissue was greater than 92% by day 14 (Table 1). More than 95% of the adherent cells expressed the markers CD73, CD90, and CD105 and exhibited weak (less than 1%) expression of the hematopoietic cell markers CD45, CD34, CD20, and CD14 (Table 1). In a previous study, after 21 days of cultivation in specialized StemPro® Differentiation Kit medium (Thermo Fisher Scientific, Waltham, MA, USA), the test culture of human adipose-derived MSCs (hAMSCs) showed positive staining for alizarin red (osteoblasts), alcian blue (chondroblasts) and oil red O (adipocytes) [26]. The minimal morphological criteria for cultured MSCs are (1) a viability of greater than 90% [27]; (2) positivity for the markers CD73, CD90, and CD105 and negativity for the blood cell markers CD45, CD34, CD20, and CD14 [19,20]; and (3) the ability to adhere to plastic and differentiate in vitro toward osteogenesis, chondrogenesis and adipogenesis [28]. Thus, the culture of hAMSCs used in this experiment satisfied the minimal morphological criteria for MSCs.

The physicochemical properties of materials affect the functional activity and differentiation of MSCs [29]. The surface roughness of the microarc CaP coating can control the osteogenic differentiation of adherent MSCs upon direct contact, as reflected in the correlations of the Ra index with cell markers of osteoblasts (ALP and OCN). In this case, the average roughness index (Ra) is closely related to the thickness and mass of the CaP coating [21], which, when degraded, releases calcium and phosphorus ions. These ions can regulate the expression of MSC markers [30]. Indeed, contact of hAMSCs with CaP-coated samples for 21 days was found to suppress the immunophenotypic traits of hAMSCs adhered to plastic as the relief of the CaP coating increased, while the population of hematopoietic cells (CD45^+^CD34^+^CD14^+^CD20^+^) increased [22].

The results shown in Table 1 indicate that a statistically significant increase (almost 2-fold) in the presentation of hematopoietic cell antigens due to the contact of the cell culture with CaP-coated titanium samples occurred by the 14th day of observation and slightly preceded the decreases in the expression of CD73, CD90, and CD105 markers.

### 2.2. In Vitro Osteogenic Differentiation

**Figure 1:**
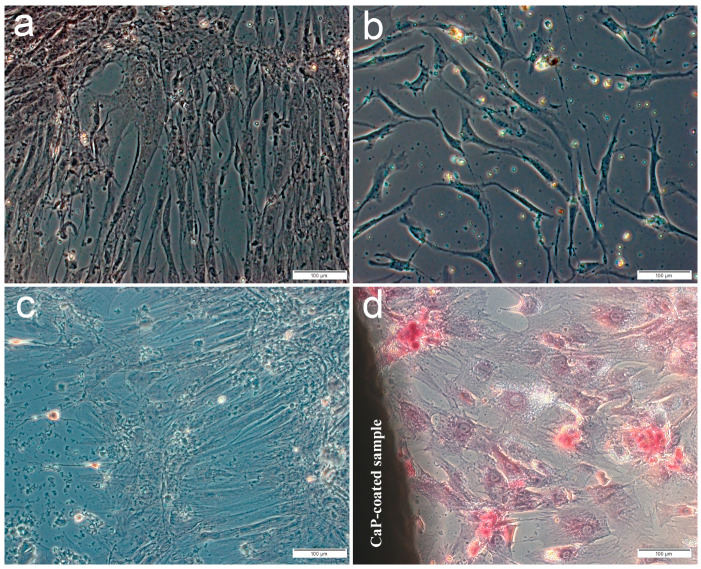
Culture of human adipose-derived mesenchymal stem cells on plastic after 14 (**a,b**) or 21 days (**c,d**) in a standard nutrient medium: **a**,**c**—control; **b**,**d**—around the microarc CaP-coated titanium sample. Staining with alizarin red S. Scale 100 µm

The main part of the plastic surface was occupied by fibroblast-like cells weakly stained by alizarin red S when hAMSCs were cultured for 14 or 21 days in a standard nutrient medium without the osteogenic supplements. Single small foci of calcification appeared in the ECM, which showed the osteogenic differentiation of individual stem cells (Figure 1a,c) including hAMSC culture after 14-day contact with the microarc CaP coating (Figure 1b). In turn, significant increase in the number and an area of mineralized nodules (Figure 1d, Table 2) indicated an initiated differentiation of hAMSCs into osteoblasts around the CaP-coated Ti substrates. 

Enhanced mineralization (calcification) of the ECM in the MSCs cultured on plastic, caused by the microarc CaP coating, was identified previously by day 21 via alizarin red staining [21,22,26], indicating in vitro the appearance of MSCs in osteoblastic hematopoietic niches [31]. Apparently, the establishment of such microterritories begins at 14 days under conditions of autocrine and paracrine secretion of chemokines as signaling molecules in the hematopoietic niches induced by the CaP coating [5].

The thickness of one side (~46 µm, Table 1 and Table 2) of the soluble microarc CaP coating allows us to consider it to be scaffold-like, and to be able to determine the behavior of hAMSCs by direct contact with the microrelief, as well as by indirect action through Ca^2+^ ions and inorganic phosphate (Pi). A wavelike dissolution/precipitation of a microarc CaP coating with a loss of more than 9% of the initial thickness was shown to occur after 8 weeks of in vitro degradation in a model biological fluid [32]. For a calcium-deficient (Ca/P ratio < 1) microarc CaP coating, the Ca^2+^ and to a greater extent Pi concentrations in model biological fluids are usually 0.5 mM per week [33] due to the balance of ion release and reverse precipitation. Both extracellular calcium and phosphorus contribute to osteogenic differentiation and mineralization of the ECM over a wide range of concentrations (0.5–16 mM for Ca^2+^; 0.09–8 mM for Pi) [8,34].

A gradient yield of Ca^2+^ and Pi was also noted for other types of CaP-containing scaffolds [35], accompanied by the formation of an alkaline microenvironment that determines the realization of the osteogenic potential of MSCs [36]. This potential can be realized through various mechanisms, including CaR activity, the activation of osteoblastic differentiation and the formation of mineralization nodules in the ECM [6]. Ca^2+^ and Pi upregulate the expression of OCN, Runx2 and BMP-2 [30]. Pi can initiate signaling via extracellular signal-regulated kinases (ERK) and cAMP/protein kinase pathways, and activation of these signaling pathways increases *BMP-2* expression [37].

### 2.3. Expression of Osteogenic, Cytokine, and Chemokine Genes

Numerous number of genes and transcription factors are necessary for the proliferation and differentiation of MSCs. Moreover, the individual knockout of each of these genes only partially changes the behavior of MSCs [9]. Therefore, for the development of regenerative medicine, the molecular basis (primarily genes and transcription factors) of the response of MSCs [10] to various stimuli must be identified.

**Figure 2:**
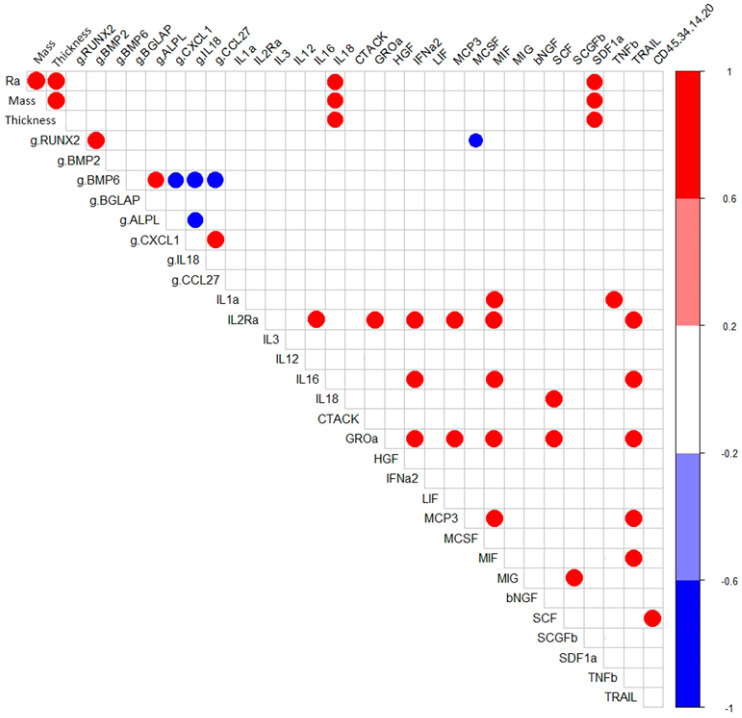
Correlations (*r* > 0.75) between the technological coating properties and in vitro indices of hAMSCs cultured for 14 days with the microarc CaP-coated titanium substrates.

Correlation analysis showed that in hAMSC cultured for 14 days around samples with a microarc CaP coating, *RUNX2*- *BMP2-* and *BMP6*- *ALPL* expressions were correlated with high coefficients (*r* = 0.96; *p* < 0.05; *n* = 7) (Figure 2). However, as shown in Table 3, statistically significant upregulation of osteogenic genes (1.4-fold that in hAMSCs cultured on plastic) was observed only for *RUNX2*, *BMP6*, and *ALPL* (Table 3). 

Notably, as indicated in Table 3, the expression of osteogenic genes began along with 7-fold increase in the transcription of *hTERT*, which regulates cell proliferation. Day 14 corresponds to the completion of the formation of three-dimensional MSC/osteoblast cell culture conditions and the beginning of the formation of mineralization nodules. 

The transcription factor Runx2 (core-binding factor subunit alpha-1=cbfa1) is considered a main determinant of osteoblast genesis from MSCs [38]. It regulates the expression of many osteoblast genes (ALP, OPN, OCN, and matrix metalloproteinase 13). Runx-driven osteogenesis is characterized by sequential expression of marker molecules (BMPs, ALP, and OCN), which alone do not always lead to mineralization of the bone matrix [12].

ALPL gene expression occurs during the early stages of osteogenesis [12]. ALP activity is necessary for subsequent mineralization of the ECM in the alkaline microenvironment [36] and appears within 7–14 days after contact with osteogenic scaffolds [35]. Ca^2+^ ions formed during degradation of biomaterials are inducers of ALP expression [35] and the differentiation of MSCs into osteoblasts is triggered through the expression of calcium-binding proteins [39]. In turn, an increase in the level of ALPL gene mRNA expression ensures the presence of free Pi in the cellular microenvironment, and this Pi binds with calcium to form CaP minerals in the bone [37].

In turn, the BGLAP gene encodes OCN, a protein that is expressed during the late stages of osteogenesis, is a marker of the terminal differentiation of MSCs into secreting osteoblasts and is produced during ECM mineralization [40]. The promoter of this gene is regulated by the transcription factor Runx2 and is activated by the BMP-2 [41].

Thus, the increased expression of the hTERT, RUNX2, BMP6, and ALPL genes but not *BGLAP* on day 14 of in vitro culture reflects a chain of events associated with the ongoing proliferation and differentiation of hAMSCs into osteoblasts as well as with the early stages of ECM mineralization. Indeed, a mineralized ECM with intense alizarin red staining was observed only by day 21 in a coculture system containing hAMSCs and a micro-arc CaP coating [5]. 

Most researchers agree that osteoblasts attach more easily to surfaces with a coarse microtopography [42,43], which may enhance osteogenic cell differentiation [44] than to those with a smooth microtopography. Independent groups of scientists revealed that increased relative mRNA expression levels of OPN, OCN [45], *RUNX2* and *ALPL* [17] are associated with osteogenic activity, and found that bone marrow MSCs cultured in vitro on rough surfaces demonstrate more intense alizarin red staining than MSCs under similar conditions on flat [17,46] and plastic [22] surfaces. These results may indicate that surface topography modulates the osteogenic differentiation of MSCs and the ECM mineralization. 

The results obtained by Sutherland et al. (2005) also indicate that cell attachment and proliferation are dependent on surface topography and that the cytoskeleton exhibits higher stress levels on coarser surfaces [47]. According to McCafferty et al. (2014), stiffness, topography, and surface chemistry can cause cytoskeletal remodeling and focal adhesion formation prior to MSC differentiation via integrin-mediated signaling pathways [48]. Cytoskeletal alterations can affect the organization and distribution of organelles and DNA, which regulate the functioning and biological activity of cells [49]. Shafrir et al. (2002) showed that microfilaments cross the nuclear pores and connect to the nuclear membrane, thereby providing a path for the transduction of signals induced by mechanical stimuli [50].

Research by McBeath and Prowse (2013) confirmed that the topography of the artificial matrix can regulate the osteogenic differentiation of MMSCs by altering the cytoskeleton [51,52]. In addition, research has suggested that the distribution of the actin cytoskeleton, in particular filamentous actin (F-actin), varies on rough surfaces [53]. The actin cytoskeleton plays an important role in the osteogenic differentiation of MSCs [54]; it is modified and changed as MSCs differentiate into osteoblasts instead of the large number of thin, parallel microfilament bundles that propagate throughout the cytoplasm in undifferentiated MSCs, thick bundles of actin filaments are located on the periphery of differentiated cells [54].

Structural alterations in the cytoskeleton lead to signal transduction to the nucleus and are associated with the activation of the nuclear transcription factors YAP (yes-associated protein 1) and TAZ (WW domain-containing transcription regulator protein 1, WWTR1) which are regulated by the actin cytoskeleton; this link explains the participation of mechanical stimuli in the osteogenic differentiation of MSCs [55,56]. Yang et al. (2016) convincingly demonstrated that different surface topographies differentially affected the activation of the transcription factors YAP/TAZ, leading to changes in the relative expression levels of osteogenic genes [17]. In turn, the transcription factors YAP/TAZ mediate the differentiation of MSCs by inducing the Runx2 coactivator, an osteoblast-specific transcription factor that affects the expression of osteogenic genes [57,58].

However, we did not find that the expression of the tested osteogenic genes in a 14-day in vitro culture of hAMSCs on plastic around samples coated by soluble microarc CaP coating was dependent on its physical parameters (roughness, weight, and thickness). The correlation coefficients of physical and biological factors varied between −0.43 and 0.52. Apparently, there are other pathways leading to activated expression of osteogenic differentiation-related genes that are not associated with CaP surface features and poor concentrations of Ca^2+^ and Pi in an intercellular medium.

Considering this possibility, the negative correlation between *RUNX2* mRNA expression and the production of the osteoclastogenic macrophage colony stimulating factor (M-CSF) (*r* = −0.79; *p* < 0.05) is interesting (Figure 2). Lienau et al. (2010) showed that hematopoietin gene expression is suppressed during chondrogenesis and endochondral ossification but not during bone remodeling [59].

In other words, in the context of the actively developing field of osteoimmunology [60], the osteogenic activity of numerous cytokines and chemokines [14] is noteworthy as a variation on the cytokine/chemokine-mediated initiation of osteogenesis, constituting an alternative to the known signaling pathways. 

### 2.4. Cytokine and Chemokine Secretion

In our study, by the 14th day of contact with CaP-coated Ti substrates the relative mRNA expression levels of the IL-18 gene and genes encoding some chemokines (CXCL1, CCL27) decreased significantly. However, *CCL7* activity remained elevated relative to that in hAMSCs cultured on plastic without CaP-coated samples (Table 3).

Secretion by hAMSCs cultured for 14 days on plastic (2D control) (Table 4; abbreviations of cytokines/chemokines are enclosed) was classified according to [61]: 

High (more than 1 ng/mL) concentration of SCGF-b;

Average (0.1–1 ng/mL) concentrations of HGF and MIF;

Low (1–100 pg/mL) concentrations of IL-2Ra; IL-3; IL-12 (p40); IL-16; IL-18; IFNα2; M-CSF; β-NGF; LIF; MCP-3 (CCL7); MIG (CXCL9); GROα (CXCL1); SCF; SDF-1α (CXCL12); TRAIL CTACK; 

Minimal (<1 pg/mL) concentrations of IL-1α and TNFβ mediators;

The inflammatory biomolecules are able to regulate osteogenesis [2,14]. In the presence of samples with a microarc CaP coating, a change in the secretory profile of hAMSCs was observed (Table 4). Specifically, the concentrations of IL-18, GROα (CXCL1) and SCF increased significantly (*p* < 0.05) (by 26%, 15% and 267%, respectively). In contrast, the levels of HGF (by 44%) and LIF (by 29%) decreased. 

During the formation of a microarc CaP coating, its thickness, controlled by the technological parameters of the microarc device, determines the mass of CaP in the coating (*r* = 0.96; *p* <0.001; *n* = 7) and the average surface roughness index (Ra) (*r* = 0.96; *p* < 0.001; *n* = 7). The tested physical properties of the microarc CaP coating (thickness, mass, and roughness) in the 14-day hAMSC culture affected the secretion of IL-18 (*r* = 0.77–0.79; *p* < 0.01; *n* = 10) and SDF- 1α (CXCL12) (*r* = 0.69–0.73; *p* < 0.02; *n* = 10) (Figure 2).

Notably, the secretion of inflammatory/migratory cytokines/chemokines was in antiphase (according to the feedback principle) with the expression of the mRNA of their genes, with the mRNA expression of the corresponding genes, with the exception of the CLEC11A gene/SCGFb pair (Table 3 and Table 4). In turn, strong inverse correlations were found between the mRNA expression level of *IL-18* with that of *ALPL* (−0.79; *p* < 0.03; *n* = 7) and *BMP6* (−0.82; *p* < 0.02; *n* = 7) and of *BMP6* with those of the chemotaxis-related genes CXCL1 (−0.79; *p* < 0.04; *n* = 7) and CCL27 (−0.82; *p* < 0.02; *n* = 7) (Figure 2). According to Cornish et al. (2003), IL-18 plays a role as an autocrine/paracrine mitogen in both osteogenic and chondrogenic cells [62] and may be a trigger of osteogenic differentiation [63].

The chemokine GROα (CXCL1), a mediator of MSC chemotaxis [64], apparently contributes to the formation of a network of inflammatory cytokines/chemokines and one of the hematopoietic stem cell (HSCs) growth factors (SCGF-b) (Figure 2). CXCL1, on the other hand, can induce osteoclastogenic activity [65]. Therefore, the molecular (gene/secretory) activity of hAMSCs in a 14-day in vitro culture (Table 3 and Table 4) may indicate the end of the proinflammatory phase and a switch to regenerative (osteogenic) processes [14], induced by the physical-chemical properties of the microarc CaP coating.

Interestingly, increasing level of IL-18 is directly correlated with an increasing concentration of SCF (*r* = 0.82; *p* < 0.03; *n* = 7), a key signaling niche molecule in HSCs [66]. Moreover, the increase in the percentage of cells expressing hematopoietic markers CD45^+^34^+^14^+^20^+^ was correlated with an increase in the SCF concentration in the hAMSC culture (*r* = 0.77; *p* < 0.05; *n* = 10) (Figure 2).

Osteoblasts form niches for HSCs including lymphoid stem cells [31]. Proinflammatory IL-18 is likely involved in the initiation of both the osteogenic differentiation of hAMSCs and the formation of osteoblastic hematopoietic niches. The low concentrations of potential inducer molecules in the total volume (1.5 mL) of the culture medium should not be confusing. The local concentrations of these factors can be extremely high near individual cells and the forming hematopoietic microterritories. Indeed, we previously noted the association of osteogenic/hematopoietic processes in cultured hAMSCs in contact with a microarc CaP coating for 14 days [5].

In mice, acute bone inflammation occurring within 7–14 days after injury triggers the subsequent phase of skeletal repair, leading to fracture healing [4]. If the timely switching of the phases of inflammation/regeneration is disrupted, including disruptions provoked by the implant, complications develop (e.g., infection, osteonecrosis, osteoporosis, pseudoarthrosis, and bone nonunion), making the prognosis significantly less favorable. Curing large bone tissue defects is a major clinical problem worldwide. Therefore, the expression of inflammatory cytokines and their receptors is of functional importance to bone remodeling, and signaling pathway modulation is a promising strategy for controlling bone regeneration [14]. However, the timing and mechanisms required for the induction of the inflammatory and regenerative reactions remain unclear.

## 3. Material and Methods

### 3.1. Samples of Naterial with a CaP Coating 

A bilateral CaP coating was formed on substrates (10 × 10 × 1 mm^3^) of commercially pure titanium (Ti, wt.%: 99.58 Ti, 0.12 O, 0.18 Fe, 0.07 C, 0.04 N, 0.01 H) by microarc oxidation (MAO) method using a Micro-Arc 3.0 system Institute of Strength Physics and Materials Science of the Siberian Branch of the Russian Academy of Sciences (ISPMS SB RAS, Tomsk, Russia) in the anode mode as described previously [33]. The electrolyte consisted of an aqueous solution of phosphoric acid (20 wt.%), calcium carbonate (9 wt.%), and synthetic hydroxyapatite (HAP, 6 wt.%).

The roughness of the test coatings was evaluated and the average roughness index (Ra) was determined using a Hommel-Etamic T1000 profilometer (Jenoptik, Jena, Germany) as described previously [67]. Ten measurements were performed for each sample. An Ra range of 2.3–4.6 μm corresponds to the biologically active range over which the relief of CaP coatings can support the osteogenic differentiation of MSCs in vivo [68].

The coating thickness was determined from cross-sectional micrographs via scanning electron microscopy (SEM; LEO EVO 50, Zeiss, Germany; Nanotech Center at ISPMS SB RAS, Tomsk, Russia). The scaffold-like CaP layer generated by MAO technique contained numerous closed and branched pores (up to 7 µm in diameter) due to the intense influence of the cascade of microarc discharges and the local electrical breakdown of the accumulating coating.

The specimens were weighed on a digital microanalytical balance (GR-202, A&D Company, Tokyo, Japan) before and after coating, and the bilateral coating mass was calculated.

Before biological testing, the samples were sterilized by dry heat in a Binder FD53 oven (Binder GmbH, Tuttlingen, Germany) at 453 K for 1 h.

### 3.2. Human Cell Isolation 

Adult hAMSCs were isolated from the lipoaspirate of a healthy man (30 years old). This study was approved by the Local Ethics Committee of Innovation Park, Immanuel Kant Baltic Federal University, Kaliningrad, Russia (permit no. 7; 9 December 2015). Informed consent for the procedure was obtained as specified previously [69]. A stromal vascular fraction and a processed lipoaspirate (PLA) with little contamination by endothelial cells, pericytes, and smooth muscle cells were obtained as described previously [70]. Subconfluent cells from the PLA were passaged five times (with each passage lasting 5–7 days) and cultured at 37 °C and 5% CO_2_ in nutrient medium consisting of 90% α-MEM (Sigma-Aldrich, St. Louis, MO, USA), 10% fetal bovine serum (Sigma-Aldrich, St. Louis, MO, USA), 0.3 g/L *L*-glutamine (Sigma-Aldrich, St. Louis, MO, USA), and 100 U/mL penicillin/streptomycin (Sigma-Aldrich, St. Louis, MO, USA) to expand the hAMSC population ex vivo.

### 3.3. Human Cell Culture

To study 14-day cell viability, gene expression, secretion, and immunophenotype, hAMSCs were cultured at a concentration of 5 × 10^4^ live cells per 1.5 mL of complete nutrient medium consisting of 90% α-MEM (Sigma-Aldrich, St. Louis, MO, USA), 10% inactivated (30 min at 56 °C) fetal bovine serum (Sigma-Aldrich, St. Louis, MO, USA), 0.3 g/L *L*-glutamine (Sigma-Aldrich, St. Louis, MO, USA), and 100 U/mL penicillin/streptomycin (Sigma-Aldrich, St. Louis, MO, USA). The nutrient medium was replaced with fresh medium every 3–4 days. One CaP-coated Ti substrate was placed in the center of the bottom of each well of a 12-well flat-bottom plate (Orange Scientific, Braine-l’Alleud, Belgium). Cells cultured without CaP-coated substrates served as 2D controls. 

### 3.4. Cytokine Assay

Supernatants from 14-day cell cultures were centrifuged for 10 min at 500 ×g. Chemokines, growth factors, and pro- and anti-inflammatory cytokines (LIF, SCF, SDF-1a, SCGF-b, M-CSF, MCP-3, MIF, MIG, TRAIL, GRO-a; IL-1a, IL-2ra, IL-3, IL-12 (p40), IL-16, IL-18, HGF, TNF-b, b-NGF, IFN-a2, and CTACK) were quantified by fluorescence flow fluorimetry using an automated Bio-Plex Protein Assay System analyzer (Bio-Rad, Hercules, CA, USA) and a commercial test system (Bio-Plex Pro Human cytokine Group II 21-Plex Panel, Bio-Rad, Hercules, CA, USA) in accordance with the manufacturer’s protocol. This cytokine/chemokine profile was previously estimated as niche signal molecules while osteoblastic differentiation of hAMSCs induced by microarc CaP-coated Ti substrates occurred in vitro [5].

### 3.5. Cellular Viability and Immunophenotype Analysis

To assess the viability, gene expression profile, and immunophenotype of hAMSCs adhered to the plastic around the CaP-coated samples, cells were preliminarily harvested with 0.05% trypsin (PanEco, Moscow, Russia) in 0.53 mM EDTA (Sigma-Aldrich, St. Louis, MO, USA) and washed twice with phosphate-buffered saline.

The surface markers on viable hAMSCs were analyzed with a Human MSC Phenotyping Kit (cat. no. 130-095-198, Miltenyi Biotec, Bergisch Gladbach, Germany), which detects the markers CD14, CD20, CD34, CD45, CD73, CD90, and CD105. After a 10-min incubation with the labeled monoclonal antibodies (mAbs), the cells were assayed using a MACS Quant flow cytometer (Miltenyi Biotec, Bergisch Gladbach, Germany) according to the manufacturer’s protocol. The in vitro viability of hAMSCs was also estimated with the MACS Quant flow cytometer (Miltenyi Biotec, Bergisch Gladbach, Germany) after staining with a solution of Annexin V: FITC (Abcam, Cambridge, UK) and propidium iodide (Abcam, Cambridge, UK) according to the manufacturer’s protocol. The flow cytometric data were analyzed using KALUZA analysis software (Beckman Coulter, Brea, CA, USA).

### 3.6. Gene Expression Analysis 

To assess the expression of osteogenic (*hTERT*, *RUNX2*, *BMP2*, *BMP6*, *BGLAP*, and *ALP*) and cytokine/chemokine (*IL-18*, *CXCL1*, *CCL27*, *CCL7*, *CXCL12*, and *CLEC11A*) genes by hAMSCs, total RNA was isolated from the obtained samples using the Extract RNA kit reagent (Eurogen, Moscow, Russia) according to the manufacturer’s protocol. Then, the isolated total RNA was reverse transcribed to cDNA using oligo(dT)23 primers (20 μM) (Beagle, Moscow, Russia) and MMLV reverse transcriptase (Eurogen, Moscow, Russia).

Multiplex polymerase chain reaction (PCR) analysis was performed in triplicate using qPCRmixHS reagents (Eurogen, Moscow, Russia), specific TaqMan probes and primers at a concentration of 10 pM (Beagle, Moscow, Russia) in a CFX96 qPCR instrument (Bio-Rad, Hercules, CA, USA). Five microliters of cDNA was used as the template, and the large ribosomal protein (RPLPO) gene was used as the reference gene.

PCR results were analyzed using the second derivative maximum method. The relative expression levels of the target genes were calculated using the modified Pfaffl formula for different amplification efficiencies. Relative quantitative analysis (relative quantification) is based on the ratio of the expression of the target gene to the expression of the reference gene and is largely sufficient for studying physiological changes in gene expression levels. The following oligonucleotide primers (Beagle, Moscow, Russia) were used (Table 5).

### 3.7. Alizarin Red Staining

Osteogenic differentiation of hAMSCs cultured on plastic around the CP-coated Ti substrates was determined as described previously [21]. To establish the self-differentiation potency of cells initiated by a microarc CaP coating, the culture medium was not saturated by osteogenic supplements. A sample with a bilateral microarc CP coating was placed in a plastic well of a 12-well flat-bottom plate (Orange Scientific, Braine-l’Alleud, Belgium). hAMSCs at a final concentration of 1.5 × 10^5^ live cells per 1.5 mL were equally seeded on and around the test samples and were cultured in complete nutrient medium described above (see item 3.3) at 100% humidity with 5% CO_2_ at 37 °C for 14 and 21 days. The medium was replaced with fresh medium every 3–4 days. Cell cultures without CaP-coated Ti substrates served as 2D controls.

Differentiated hAMSCs were stained with 2% alizarin red S (ARS, Sigma-Aldrich, St. Louis, MO, USA) to identify mineralization of the extracellular matrix produced by osteoblasts. All staining procedures were performed as recommended by the manufacturer. The results were assessed with a Zeiss Axio Observer A1 microscope (Carl Zeiss Microscopy, LLC, Thornwood, NY, USA) using ZEN 2012 software (Carl Zeiss Microscopy, LLC) on plastic surfaces around the CP-coated Ti substrates. The average areas of ARS staining (in mm^2^) were calculated on digital images via quantitative computer morphometry with the help of ImageJ v. 1.43 software in each of 3–4 wells per test group.

### 3.8. Statistical Analysis

Descriptive statistical methods, as well as hypothesis testing methods implemented in the standard STATISTICA for Windows 13.3 packages, were used to analyze the obtained data. The data were tested for normality using the Kolmogorov–Smirnov criterion. The following distribution parameters were calculated: the median (Me) and the 25% (Q1) and 75% (Q3) quartiles. To assess the statistical significance of the differences, the nonparametric Mann–Whitney U-test was used. The relationship between the studied parameters was established by correlation analysis (*r*). Differences were considered statistically significant at a significance level of *p* < 0.05.

## 4. Conclusions

**Figure 3:**
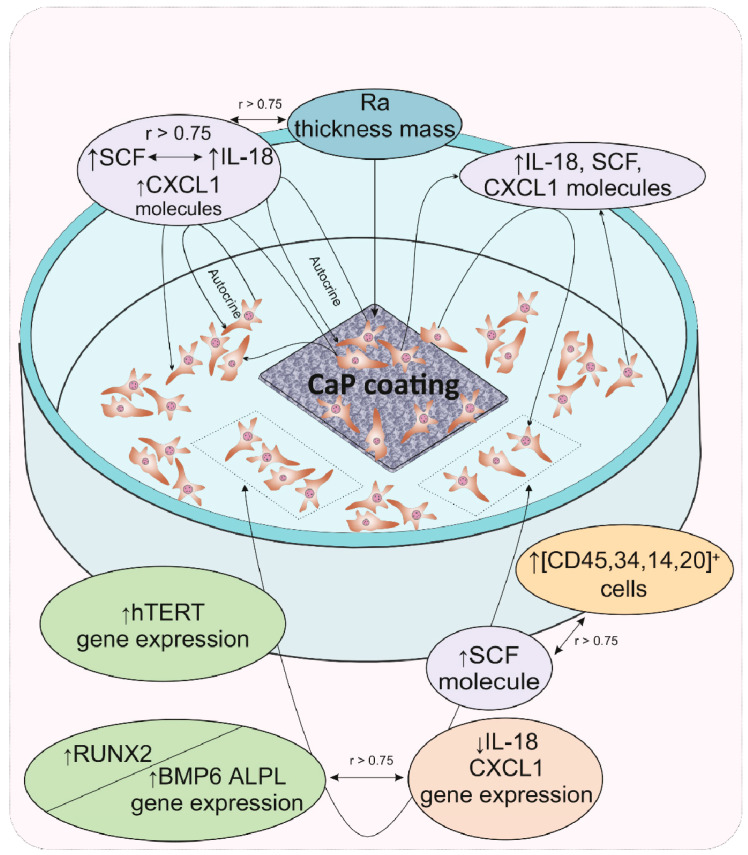
Schematic representation summarizing the main results of gene expression regulation in and secretory activity of mesenchymal stem cells after 14 days of in vitro contact with the microarc CaP coating on a titanium substrate. Abbreviations are presented in the text of the article and in Table 4.

The results of cultural and correlation analyses in our model experiment showed that for hAMSCs, the 14th day of in vitro culture in contact with a rough microarc CaP coating is a checkpoint marking the end of the proinflammatory phase, the completion of hAMSC proliferation accompanied by the formation of three-dimensional MSC culture conditions, and the beginning of osteoblastic differentiation. The increasing secretion of pleiotropic inflammatory biomolecules by the feedback principle inhibits the expression of the corresponding genes, which may be the key step in an additional (relative to the already known) mechanism of initiating the expression of osteogenic genes. With the expanding network of cytokines and chemokines with osteomodulatory properties (IL-18, CXCL1), as well as the functions of signaling molecules in hematopoietic niches (e.g., SCF), HSC microterritories are established. According to correlation analysis, the described cellular-molecular changes may be induced by the properties of the microarc CaP coating (roughness, primarily for cells on the CaP surface), calcium and phosphorus ions secreted by the coating, and, to a greater extent, autocrine and paracrine cytokines/chemokines secreted by hAMSCs. A schematic representation summarizing our general results is shown in Figure 3.

To further develop the scientific foundations of bone tissue bioengineering, the differential significance of the relief versus the solubility of microarc CaP coatings for the epigenomic regulatory effects on MSCs must be determined. The knowledge gained can significantly improve the results of personalized osteosynthesis using the microarc CaP-coated Ti substrates [23] to treat bone tissue injuries and diseases.

## Figures and Tables

**Table 1:** Viability and immunophenotype of human adipose-derived MSCs (hAMSCs) collected from plastic after 14 days of coculture with the microarc CaP-coated titanium substrates, Me (Q1–Q3).

Parameters of One Surface of a Bilateral CaP Coating on a Titanium Substrate, *n* = 7			Number of Viable Cells %	Number of Apoptotic cells %	Number of Necrotic cells %	Stem Cell Markers, %			Hemato-poietic Cell Markers%
Ra µm	Thickness µm	Weight mg				CD73	CD90	CD105	[CD45,34,14,20]
Mesenchymal stem cell (MSC) culture on plastic (2D control), *n* = 3 ^1^									
−	−	−	92.54 (91.02–95.39)	1.54 (0.84–2.68)	5.02 (4.01–6.3)	95.31 (91.74–95.73)	98.57 (98.09–98.79)	98.86 (98.66–99.14)	0.36 (0.33–0.37)
hAMSC culture on plastic in contact with the CaP-coated titanium substrates									
3.1 (2.4–3.3)	46.0 (35–53.5)	13.0 (11.2–14.0)	91.77 (91.47–93.14)	3.22 (1.43–4.20)	4.33 (3.64–6.8)	93.70 (85.71–93.73)	96.80 (92.83–97.31)	97.05 (93.60–97.74)	0.63 ^2^ (0.56–0.63)

^1^*n*-the number of tested samples (wells); each measurement was done in triplicate; ^2^ Statistical difference (Pu < 0.05) is shown according to Mann–Whitney U-test. (−) the measurements were not done on plastic surface.

**Table 2:** Osteogenic differentiation of hAMSCs on plastic after 21 days of in vitro coculture with the microarc CaP-coated titanium substrates, Me (Q1–Q3).

Parameters of One Surface of a Bilateral CaP Coating on a Titanium Substrate, *n* = 3			Indices of Alizarin Red S Staining	
Ra µm	Thickness µm	Weight mg	The Number of the Sites of Cell Culture Mineralization per Well	An Average Area of the Mineralization Sites, mm^2^
MSC culture on plastic (2D control), *n* = 4 ^1^				
−	−	−	0	0
hAMSC culture on plastic in contact with the CaP-coated titanium substrates				
2.49 (2.20; 2.90)	45.5 (33.0; 56.5)	11.8 (9.6; 13.8)	156 (155–208) ^2^	0.0037 (0.0034–0.0043) ^2^

^1^*n*-the number of tested samples (wells); ^2^ Statistical differences (Pu < 0.05) are shown with the 2D control according to the Mann–Whitney U-test. (−) the measurements were not done on plastic surface.

**Table 3:** Relative gene expression levels (fold) in hAMSCs collected from plastic after 14 days of in vitro coculture with the microarc CaP-coated titanium substrates, Me (Q1; Q3).

Parameters of One Surface of a Bilateral CaP Coating on a Titanium Substrate *n* = 7 ^1^			Relative Expression of Osteogenic Genes					
Ra µm	Thickness µm	Weight mg	*hTERT*	*RUNX2*	*BMP2*	*BMP6*	*BGLAP*	*ALPL*
3.1 (2.4; 3.3)	51.5 (37.0; 53.5)	13.1 (11.0; 14.4)	6.9 ^2^ (6.4; 56.5)	1.44 ^2^ (1.07; 1.69)	1.05 (−1.40; 1.57)	1.40 ^2^ (1.04; 1.69)	−0.92 (−1.41; 1.69)	1.43 ^2^ (1.03; 1.63)
			Relative expression of cytokine and chemokine genes					
			*IL-18*	*CXCL1*	*CCL27*	*CCL7*	*CXCL12*	*CLEC11A*
			−1.47 ^2^ (−3.52; −1.22)	−1.16 ^2^ (−1.24; −1.13)	−3.10 ^2^ (−3.28; −1.84)	3.11 ^2^ (2.33; 3.51)	2.24 (−3.52; 4.31)	−2.10 (−2.44; 3.79)

^1^*n*-the number of tested samples; ^2^*p* < 0.05 compared with cell culture without test samples according to the Mann–Whitney U-test; (−) sign means inhibition of relative gene expression in comparison with a control cell culture on plastic without samples; *hTERT*-gene of human telomerase reverse transcriptase; *BGLAP*-gene of bone gamma-carboxyglutamate protein (OCN).

**Table 4:** Secretory activity (pg/mL) of hAMSCs after 14 days of culture with the microarc CaP-coated titanium substrates, Me (Q1-Q3).

Factors		hAMSC Culture on Plastic, *n* = 3	hAMSC Cultures in Contact with the CaP-coated Titanium Substrates, *n* = 7
Surface parameters of a CaP coating	Ra, µm	0	3.1 (2.4–3.3)
	Thickness, µm	0	46.0 (35–53.5)
	Weight, mg	0	13.0 (11.2–14.0)
Inflammatory interleukins and cytokines	IL-1α	0.68 (0.65–1.10)	0.77 (0.69–0.85)
	IL-2Ra	17.84 (10.23–27.63)	8.48 (8.48–12.8)
	IL-3	5. 54 (2.51–6.01)	3.29 (2.38–7.54)
	IL-12 (p40)	14.62 (14.62–54.73)	38.79 (12.78–52.11)
	IL-16	33.19 (21.52–47.69)	18.22 (15.87–26.61)
	IL-18	6.40 (3.43–7.21)	8.09 * (7.53–11.13)
	TNFβ	0.49 (0.42–0.82)	0.55 (0.21–0.57)
	IFNα2	17.10 (17.10–18.41)	16.54 (15.91–17.45)
Growth factors	M-CSF	15.55 (13.47–19.80)	18.20 (11.42–20.03)
	β-NGF	8.21 (6.55–8.61)	7.47 (5.13–8.33)
	HGF	321 (299–413)	178 * (162–192)
Chemokines	LIF	14.43 (11.03–16.03)	10.30 * (9.33–10.32)
	MCP-3(CCL7)	95.57 (45.26–112.16)	60.18 (57.82–66.99)
	MIF	536 (405–966)	492 (254–667)
	MIG (CXCL9)	12.16 (8.97–12.80)	8.97 (8.97–14.06)
	GROα (CXCL1)	36.77 (35.68–38.92)	42.34 * (39.21–49.13)
	SCF	3.14 (2.18–4.45)	11.49 * (6.82–11.59)
	SCGF-b	12838 (11657–13045)	11629 (9997–15633)
	SDF-1α (CXCL12)	81.84 (43.22–101.84)	70.37 (49.40–76.90)
	TRAIL	4.04 (3.04–4.37)	3.21 (2.71–4.20)
	CTACK (CCL27)	34.49 (32.30–43.92)	40.40 (40.40–58.65)

* Each measurement was done in triplicate. IL—interleukin; TNFβ—tumor necrosis factor beta; IFNα2—human interferon α2; M-CSF—monocyte colony stimulating factor; β-NGF—beta-nerve growth factor; HGF—hepatocyte growth factor; LIF—leukemia inhibitory factor; MCP-3—monocyte chemotactic protein-3—chemokine (C-C motif) ligand 7 (CCL7); MIF—macrophage migration inhibitory factor; MIG—monokine induced by gamma interferon—chemokine (C-X-C motif) ligand 9 (CXCL9); GROα—growth regulated oncogene-alpha—chemokine (C-X-C motif) ligand 1 (CXCL1); SCF—stem cell factor; SCGFb—stem cell growth factor beta (C-type lectin domain family 11 member A; CLEC11A) beta; SDF-1α—stromal derived factor 1 alpha—C-X-C motif chemokine 12 (CXCL12); TRAIL—tumor necrosis factor ligand superfamily member 10 (TNF-related apoptosis-inducing ligand); CTACK—Chemokine C-C motif ligand 27.

**Table 5:** Sequences of oligonucleotide primers used in the experiment.

*RUNX2*_for 5′-CCAGAAGGCACAGACAGAAG-3′
*RUNX2*_rev 5′-GATGAGGAATGCGCCCTAAA-3′
*BMP2*_for 5′-ACGAGGTCCTGAGCGAGTT-3′
*BMP2*_rev 5′-GACCTGAGTGCCTGCGATAC-3′
*BMP6*_for 5′-TTACAGGAGCATCAGCACAG-3′
*BMP6*_rev 5′-GGAGTCACAACCCACAGATT-3′
*BGP*_for5′- GAGGGTATAAACAGTGCTGGAG-3′
*BGP*_rev5′-AATAGGGCGAGGAGTGTGA-3′
*ALP*_for5′-GGGAAATCTGTGGGCATTGT-3′
*ALP*_rev5′-GAGTACCAGTCCCGGTCAGC-3′
*RPLPO*_for 5′-GGCGACCTGGAAGTCCAACT-3′
*RPLPO*_rev 5′-CCATCAGCACCACAGCCTTC-3′
*RUNX2*_probe 5′-FAM-AGTTTGTTCTCTGACCGCCTCAGT--BHQ1-3′
*BMP2*_probe 5′-FAM-CTGAAACAGAGACCCACCCCCAGCA-BHQ1-3′
*BMP6*_probe 5′-FAM-CCTCAGAAGAAGGCTGGCTGGAAT-BHQ1 -3′
*BGP*_probe 5′-FAM-CAGCCACCGAGACACCATGAGA-BHQ1-3′
*ALP*_probe 5′-FAM-ACCACGAGAGTGAACCATGCCA-BHQ1-3′
*RPLPO*_probe Bgl635-5′-ATCTGCTGCATCTGCTTGGAGCCCA-3′-BHQ-2
